# Animal Models of Diabetic Macrovascular Complications: Key Players in the Development of New Therapeutic Approaches

**DOI:** 10.1155/2015/404085

**Published:** 2015-02-15

**Authors:** Suvi E. Heinonen, Guillem Genové, Eva Bengtsson, Thomas Hübschle, Lina Åkesson, Katrin Hiss, Agnes Benardeau, Seppo Ylä-Herttuala, Ann-Cathrine Jönsson-Rylander, Maria F. Gomez

**Affiliations:** ^1^Bioscience, Cardiovascular and Metabolic Diseases, Innovative Medicines and Early Development, AstraZeneca R&D, 43183 Mölndal, Sweden; ^2^Division of Vascular Biology, Department of Medical Biochemistry and Biophysics, Karolinska Institutet, 17177 Stockholm, Sweden; ^3^Department of Clinical Sciences, Lund University Diabetes Centre (LUDC), Lund University, 20502 Malmö, Sweden; ^4^R&D Diabetes Division, Translational Medicine, Sanofi-Aventis, 65926 Frankfurt am Main, Germany; ^5^Department of Biotechnology and Molecular Medicine, A.I. Virtanen Institute for Molecular Sciences, University of Eastern Finland, 70210 Kuopio, Finland; ^6^Pharmaceutical Division, pRED, CV and Metabolic Disease, Hoffmann-La Roche, 4070 Basel, Switzerland

## Abstract

Diabetes mellitus is a lifelong, incapacitating metabolic disease associated with chronic macrovascular complications (coronary heart disease, stroke, and peripheral vascular disease) and microvascular disorders leading to damage of the kidneys (nephropathy) and eyes (retinopathy). Based on the current trends, the rising prevalence of diabetes worldwide will lead to increased cardiovascular morbidity and mortality. Therefore, novel means to prevent and treat these complications are needed. Under the auspices of the IMI (Innovative Medicines Initiative), the SUMMIT (SUrrogate markers for Micro- and Macrovascular hard end points for Innovative diabetes Tools) consortium is working on the development of novel animal models that better replicate vascular complications of diabetes and on the characterization of the available models. In the past years, with the high level of genomic information available and more advanced molecular tools, a very large number of models has been created. Selecting the right model for a specific study is not a trivial task and will have an impact on the study results and their interpretation. This review gathers information on the available experimental animal models of diabetic macrovascular complications and evaluates their pros and cons for research purposes as well as for drug development.

## 1. Introduction

In patients with diabetes, a much more widespread and aggressive form of atherosclerosis is observed in the coronary arteries, lower extremities, and extracranial carotid arteries, causing nearly 80% of all deaths and much of the disability in these patients. Both type 1 (T1D) and type 2 diabetes (T2D) are independent risk factors for myocardial infarction, peripheral vascular disease, and stroke [1, 2]. The pathophysiological mechanisms underlying this accelerated and aggravated atherosclerosis are complex and a matter of intense research (reviewed elsewhere [3]) but are driven by the metabolic changes that take place in diabetes, including hyperglycemia, insulin resistance, and elevated free fatty acid production. Apart from accelerated atherosclerosis, diabetic patients have increased risk of cardiac dysfunction and heart failure [4]. In this review we will discuss the value and limitations of some of the animal models of macrovascular disease available today, with focus on atherosclerosis and diabetic cardiomyopathy (DCM). We will also consider the translational value of the current animal models, that is, if they can be used to predict the impact of interventions in clinical trials.

## 2. Pros and Cons of Available Models

Until the early nineties, atherogenesis was studied mainly in primates and in low-density lipoprotein (LDL) receptor-deficient rabbits. With the advances in genetic engineering, mice became soon the preferred species for atherosclerosis studies. However, the production of knock-out rats has recently become economically and technically feasible and although the area is in its infancy, rat models can be anticipated to increase in popularity. Compared to humans, rodents are in general very resistant to the development of atherosclerosis, primarily due to differences in lipoprotein metabolism [5]. Rodents lack the cholesteryl ester transfer protein (CETP), which plays a central role in lipoprotein metabolism by exchanging cholesteryl esters with triglycerides. In humans, a genetic deficiency of CETP leads to increased HDL cholesterol and an antiatherogenic state [6]. Thus, on a normal chow diet, rodents have low levels of plasma cholesterol (<2.5 mmol/L) mostly contained in the antiatherogenic high-density lipoprotein (HDL) fraction and hence do not develop plaques. Even after being fed with very high fat diets for a long time, animals only develop early signs of atherosclerosis, unless further genetic modifications are introduced. These and other basic physiological differences between human, mouse, and rat, of relevance for the study of vascular complications, are summarized in Table 1. Apart from obvious differences in vessel diameter, the site preferences for lesion development, mainly determined by the hemodynamic forces experienced by the endothelium, are different in humans and rodents (reviewed in [7]). For example, compared to humans, the blood flow in the murine aortic sinus is much more disrupted due to the very rapid heart rate (>400 and up to 550 bpm in mice versus 60–80 bpm in humans) [8]. So while the vast majority of atherosclerosis studies performed in murine models focus on the aortic sinus, this is not a site typically involved in human atherosclerosis.

Early studies also revealed major differences in the susceptibility to develop plaque depending on the inbred strain used, explaining why C57BL/6J mice are by far the most used strain in the atherosclerosis field and BALBC/J a less favored one [9]. The genetic background is also critical for the development of hyperglycemia, with C57BL/6 mice being one of the most vulnerable when compared to 5 inbred strains (DBA/2 > C57BL/6 > MRL/Mp > 129/SvEv > BALBC) [10, 11]. However, C57BL/6 mice are relatively resistant to diabetic nephropathy [12], which makes it difficult to use the same model for both macro- and microvascular disease assessment.

### 2.1. Dyslipidemic Backgrounds

The apolipoprotein E (ApoE) deficient (ApoE^−/−^) mouse was one of the first atherosclerosis models developed [13]. Mice have delayed clearance of lipoproteins and even when fed normal chow diet, their cholesterol levels increase 4-fold, leading to the development of atherosclerotic lesions [14]. However, lack of ApoE results in accumulation of chylomicron and very low-density lipoprotein (VLDL) remnants, a lipoprotein profile very different from humans [15]. Somewhat milder and widely used models of hyperlipidemia include mice expressing a mutant form of ApoE, the ApoE^*^3-Leiden [16], and mice deficient in LDL receptor (LDLR^−/−^) [17], which require dietary cholesterol supplementation for atherosclerosis development. To create more human-like lipoprotein profiles, LDLR^−/−^ mice that exclusively synthetize ApoB100 (LDLR^−/−^ApoB^100/100^ and LDLR^−/−^Apobec-1^−/−^ mice) have been generated by inhibiting the synthesis of apolipoprotein B48 (ApoB48) either by targeted mutations [18] or by preventing the enzymatic editing of ApoB mRNA [19, 20]. These last two models, together with a modified version of the ApoE^*^3-Leiden mice that expresses human cholesteryl ester transfer protein (CETP) [21], best recreate the human lipoprotein profile and should be considered as first hand options for studies requiring a dyslipidemic background.

### 2.2. Atherosclerosis in Diet-Induced Diabetic Models

The development of animal models that mimic both the metabolic abnormalities of diabetes and a macrovascular phenotype has been challenging. One widely used model is the diet-induced obese (DIO) C57Bl/6J mouse, which develops obesity, moderate diabetes, and a 2-fold increase in plasma lipoprotein levels, but only small lipid deposits in the aortic sinus of 40% of the mice after 14 weeks of diabetogenic diet [22].

Along these lines, studies performed by this consortium to evaluate the effects of a combined high fat/high sugar diet and of aging on vascular and renal function demonstrated that male DIO C57BL6J mice had an early obesity onset after ~10 weeks of diet and developed fasting hyperinsulinemia and hyperglycemia and pronounced insulin resistance and glucose intolerance after ~15 weeks of diet (Benardeau et al., unpublished). Mice developed hepatic and renal lesions that progressed to nonalcoholic steatohepatitis (NASH) and early changes in the kidney histology (glomerular hypertrophy, mesangial expansion, glomerulosclerosis, and inflammation) after ~30 weeks of diet. However, despite the long dietary challenge and their advanced age, DIO mice failed to develop significant changes in the vasculature (or in the kidney). Even when ApoE^−/−^ and LDLr^−/−^ mice were fed high fat diet for 12 months, no vascular phenotype was observed despite extreme dyslipidemia and generalized lipid accumulation, suggesting that the DIO mice are poor models for the study of complications.

There is also large variation in the diet effects depending on the model. As summarized in Table 2, several models develop atherosclerosis after diet manipulations, despite inconsistent effects on blood glucose or insulin levels or on the development of insulin resistance. Overall, diabetic features induced by diet tend to be so subtle that the observed increases in atherosclerosis are likely due to hyperlipidemia rather than alterations in glucose metabolism.

### 2.3. Atherosclerosis in Chemically or Genetically Induced Diabetic Models

Alternative approaches to the diet have been the use of chemical toxins or additional genetic manipulation for the induction of diabetes in dyslipidemic atherosclerosis-prone strains. Induction of diabetes with streptozotocin (STZ) in different atherosclerosis models, and especially in ApoE^−/−^ mice, has been widely used to study the effects of therapeutic agents or the mechanisms of accelerated atherosclerosis (reviewed in [23]). STZ is a toxic glucose analogue, which accumulates in pancreatic *β*-cells and causes cell death leading to insulinopenic diabetes with hyperglycemia. Although resembling type 1 diabetes, using STZ does not fully replicate it since it usually does not induce ketosis and the treated mice do not require insulin therapy. Nevertheless, the use of chemical toxins effectively results in acute hyperglycemia and has several advantages: protocols are standardized; onset of diabetes is rapid and can be triggered at different phases of lesion development. However, STZ can also produce nonspecific toxicity, especially if a single high-dose injection is used, but also if safer low-dose STZ regimes are used, therefore caution is recommended for interpretation of data obtained close in time to the STZ injections.

Toxicity can be circumvented by the complementary use of more chronic, genetically modified models, such as the recently developed Ins2^AKITA^ (or Akita) mouse on ApoE^−/−^ [24] or LDLR^−/−^ [25, 26] backgrounds. Akita mice have a mutation on the insulin 2 gene (Cys96Tyr), leading to a misfolding of the proinsulin 2 protein, endoplasmic reticulum stress, nonspecific impairment of secretory pathways, and beta cell apoptosis, all resulting in T1D. Severe irreversible hyperglycemia (20 mmol/L throughout life, more prominent in male mice), hypoinsulinaemia, and polydipsia at 3-4 weeks of age are central features of the model, which replicates retinopathy [27], nephropathy [28, 29], and neuropathy [30] and is associated with increased oxidative stress and premature senescence [10, 31]. Akita mice cross-bred to ApoE^−/−^ [24] and LDLR^−/−^ [25, 26] backgrounds have 2-3-fold increased atherosclerosis and enhanced accumulation of macrophages and T-cells in plaques when compared to nondiabetic control mice, as well as concomitant increases in non-HDL cholesterol and triglyceride levels. Nevertheless, this model provides a versatile tool for the study of diabetic complications in the context of hyperglycemia.

Studies based on these two different methods to induce diabetes (chemical toxins or genetic manipulation) and their outcomes with respect to plasma lipid levels and plaque development are summarized in Tables 3 and 4.

### 2.4. Animal Models with Combined Risk Profiles

Although hyperglycemia is a strong and recognized risk factor for micro- and macrovascular complications in patients with T1D [32–34], in T2D patients, hyperglycemia is primarily associated with microvascular complications [35]. Instead, a cluster of cardiovascular risk factors seem to drive macrovascular disease in T2D. Consequently, efforts have been made to create animal models that exhibit combined risk profiles. Examples of such models are the leptin deficient (*ob/ob*) or leptin receptor-deficient (*db/db*) mice on ApoE^−/−^ or on LDLR^−/−^ backgrounds [36–38] or on combinations thereof (i.e., ApoE^−/−^ApoB^100/100^ and LDLR^−/−^ApoB^100/100^ [39]). Also the agouti (*A* ^*y*^) mouse, a polygenic model of obesity-induced diabetes, has been combined with hyperlipidemic strains [40, 41].

One of the major limitations of these models is the fact that diabetes is often accompanied by large increases in plasma lipid levels, which is not a feature of the human disease and makes it very difficult to dissect the individual contributions of hyperglycemia and hyperlipidemia to atherosclerotic plaque formation. Also, despite having a complex metabolic profile (e.g., obesity, T2D, altered carbohydrate and fat oxidation rates, disturbed circadian rhythm, and higher serum calcium phosphorous product) and a 3-4-fold increase in aortic atherosclerosis, ApoE^−/−^ *db/db* mice show no dramatic increase in arterial stiffness, as assessed by measurements of pulse wave velocity (Hübschle T, unpublished data). Only when stiffness index *β* was calculated by normalization to diastolic blood pressure, differences were detected in males (*P* = 0.0008) but only trends were observed in females (*P* = 0.055, *n* = 12–16). Considering that arterial stiffness has independent predictive value for CVD in diabetic patients, the translational value of this model may be questionable.

There are only a handful of models where diabetes induction does not evoke major lipid changes. One such model is ApoE^−/−^ mice heterozygous for insulin receptor substrate (IRS) 2 [42]. On a high fat diet, both IRS2^+/−^ApoE^−/−^ and IRS2^−/−^ApoE^−/−^ mice display glucose intolerance, hyperinsulinemia, and increased aortic lesion development when compared to IRS2^+/+^ApoE^−/−^, but unchanged plasma lipid levels [43]. Accelerated atherosclerosis is also seen in a recent model of ApoE^−/−^ mice heterozygous for insulin receptor and insulin receptor substrate 1, in which hyperinsulinemia and insulin resistance promote vascular dysfunction and inflammation, leading to increased lesion development [44]. Another model currently under development by this consortium is the ApoE^−/−^ mouse heterozygous deficient for glucokinase, which shows a promising phenotype with stable hyperglycemia without changes in lipid levels (Heinonen et al., unpublished). However, whenever using the ApoE^−/−^ mice, the nonhuman lipoprotein profile may become a disadvantage. Therefore, diabetic models with a more human-like lipoprotein profile, such as the LDLR^−/−^ApoB^100/100^ mice overexpressing insulin-like growth factor II (IGF-II), may be preferred. These mice develop calcified, advanced, and more complex atherosclerotic lesions with plasma lipid levels identical to those of nondiabetic control LDLR^−/−^ ApoB^100/100^ mice [45].

### 2.5. Models That Develop Coronary Artery Disease and Myocardial Infarction

Most studies of diabetic macrovascular complications focus on the development of atherosclerosis, while less is known about other complications such as coronary artery disease and myocardial infarction. These have been extensively examined in nondiabetic hypercholesterolemic models [14, 46–53], but data in the context of diabetes is sparse. In the IGF-II transgenic LDLR^−/−^ApoB^100/100^ mice the status of coronary artery atherosclerosis and its relation to cardiac function were found to be similar to nondiabetic LDLR^−/−^ApoB^100/100^ mice [54]. In STZ-induced diabetic ApoE^−/−^ mice, impaired capillarization and perfusion of the ischemic hindlimb has been reported [55]; however, when arteriogenesis was compared in mouse models of T1D (STZ-induction and the NOD mice), insulin resistance (*ob/ob*), and hypercholesterolemia (ApoE^*^3-Leiden mouse), hypercholesterolemia was found to be by far the more effective stimulus for reducing collateral arterial growth [56].

Although accelerated atherosclerosis plays a major role in the increased cardiovascular mortality in diabetes, epidemiological data indicates that diabetes increases the risk of cardiac dysfunction and heart failure independently of coexistence of coronary artery disease, hypertension, or other macrovascular risk factors [4]. Diabetic cardiomyopathy (DCM) describes the functional (primarily diastolic dysfunction) and structural (hypertrophy) changes that occur in the left ventricle, leading to heart failure. Because of the natural resistance of mice to atherosclerosis, diabetic mouse models provide a convenient tool for studying DCM. The proposed criteria for a “good” DCM model in the mouse [57] are (1) evidence of left ventricular and/or diastolic dysfunction, (2) interstitial or replacement fibrosis, and (3) hypertrophy of the left ventricle. To date, available models present varying phenotypes with left ventricular diastolic dysfunction being the dominant feature in the Akita mice [58] and systolic dysfunction in OVE26 [59], NOD [60], and STZ treated mice (reviewed in [57, 61]). In the* ob/ob* and* db/db* mice the most common finding is cardiac hypertrophy, with contractile disturbances and sometimes increased chamber stiffness [61]. In general, it seems that hyperglycemia and the absence of insulin are sufficient to cause functional defects but the structural changes of DCM are more difficult to replicate in animal models. Moreover, while in humans the clinical picture of DCM is similar in T1D and T2D, mouse models of T2D usually have preserved systolic function in contrast to T1D models.

### 2.6. Rat Models of Macrovascular Disease

While mice have dominated the scene for studies of diabetes-induced atherosclerosis, ApoE and LDLr knockout rats, as well as leptin deficient rats, have recently become commercially available (SAGE Labs). Even though there is not yet enough data to comment on their value, these new models seem promising.

In general, rat models of diabetes have proven resistant to the development of macrovascular complications but useful for studies of microvascular complications. One example is the BioBreeding (BB) rat, which is the most widely used model for the study of T1D. More or less inbred BB strains display varying incidence (70–100%) and age of diabetes onset (40–200 days). The effects of medium to longstanding diabetes in the BB rat are limited to mild effects on the myocardium with progressive loss of myofilaments [62], contractile dysfunction in the perfused heart [63], and impaired angiogenic sprouting of the aorta [64]. Given the mild phenotype, it is indeed tempting to suggest the use of BB rats for dissecting genes conferring resistance to vascular complications.

Regarding rat models of T2D or models with combined risk profiles, several strains have been developed and extensively studied. Most of these strains are obese and derive from either the Zucker fatty (fa/fa) rat described by L. M. Zucker and T. F. Zucker [65] or the Corpulent strains (cp/cp) described by Koletsky [66], having either leptin receptors with 10-fold lower affinity for leptin or complete lack of leptin receptor activity. Zucker fatty rats are insulin resistant, hyperlipemic, and hyperinsulinemic but are not prone to develop atherosclerosis. Zucker diabetic rats (ZDF) were derived from fatty male rats that displayed hyperglycemia by selective inbreeding of this trait. Despite an early onset of diabetes (~10 weeks of age), ZDF rats do not develop atherosclerosis or macrovascular complications but have proven useful for studies of microvascular complications and diabetic nephropathy, as well as for testing various treatment strategies, including traditional insulin sensitizing agents and anti-inflammatory and vasoregulatory drugs [67–70].

The Koletsky mutation has been transferred to a number of different genetic backgrounds, resulting in various phenotypes. One being the Jcr:LA-*cp* rat, which results from the partial backcross into the standard LA/N strain and can be considered as a metabolic syndrome model. Rats are obese, hyperlipidemic, and insulin resistant but normotensive. At nine months of age, male Jcr:LA-*cp* rats have both myocardial and early atherosclerotic lesions, which evolve into more mature plaques at 20 months of age [71]. CP rats have also been used to test treatments for diabetic complications, most studies focusing on agents or diets affecting lipid metabolism [72]. Studies using Jcr:LA-*cp* rats have given new insights into the overproduction of intestinal chylomicrons under conditions of insulin resistance and into their contribution to atherogenesis [73]. One drawback is the advanced age required for the animals to develop complications. Introgression of the Koletsky mutation into other genetic backgrounds may be required to accelerate disease progression.

Another well-established rat model of diabetes is the Goto-Kakizaki (GK) Rat, which originates from the Wistar rat by selected breeding of animals with reduced glucose tolerance. GK rats are not obese but develop T2D early in life mainly due to impaired insulin secretion rather than insulin resistance. In this model, mild hyperglycemia promotes resistance artery remodeling resulting in increased medial thickness [74], impaired myogenic tone in cerebral and coronary arteries [75], and a generalized endothelial dysfunction [76], but no atherosclerosis.

### 2.7. Noninvasive Novel Methods for Monitoring Atherosclerosis* In Vivo*

Plaque burden or plaque size is the preferred primary readout in animal models as in humans and is usually histologically determined at the end of experimental studies. However, recent advances now allow the measurement of plaque progression/regression noninvasively in rodents using ultrasound. This technique enables longitudinal studies and better statistical power, reducing the number of animals required. At termination, complementary evaluations of plaque area and composition at the same anatomical sites using histology can be performed. Recent studies have shown a very good correlation between the histological and the noninvasive measurements of plaque size in the brachiocephalic artery, aorta, and carotid arteries [77, 78].

Another noninvasive method now technically feasible in rodents is the measurement of coronary flow velocity reserve (CFVR). Left coronary artery blood flow is measured using color Doppler guided echocardiography and a high-frequency ultrasound biomicroscope at normal flow and after hyperemic dilation (i.e., with adenosine) [79]. CFVR has been shown to be a strong prognostic parameter for hard cardiovascular events in many patients groups [80] and to be reduced in LDLR^−/−^ [81], LDLR^−/−^ApoB^100/100^ [53], and ApoE^−/−^ mice [79] as well as in db/db mice [82] and could thus be used as an end-point in studies evaluating the effects of novel therapeutic agents.

## 3. Can the Available Animal Models Reliably Predict the Effect of Interventions in Clinical Trials?

New drug candidates often fail in the clinical phase due to insufficient efficacy not anticipated from the preclinical* in vivo* studies. This translational failure could be partly explained by shortcomings in the design of clinical trials or by overoptimistic conclusions obtained from methodologically flawed animal studies or by the fact that the models do not sufficiently reflect disease as it is seen in humans (and combinations thereof, reviewed in [83]). The first steps towards increased drug approval rate are the identification of key issues contributing to poor translation and an increased focus on “backtranslational” studies (from humans to animals) for more robust validation of the models. In the following paragraphs we discuss how standard-of-care antidiabetic and lipid-lowering therapies backtranslate to available animal models.

### 3.1. Cardiovascular Effects of Antidiabetic Agents

For decades, new diabetes drugs were approved primarily based on their glucose-lowering efficacy. However, after reports of increased cardiovascular risk with muraglitazar [84] and rosiglitazone [85], regulatory agencies compiled new guidelines to ensure that novel drugs would be at least neutral with regard to cardiovascular effects [86, 87] and future treatments will certainly favor compounds having beneficial cardiovascular effects. These new regulations should be taken into account in preclinical studies also, as more accurate prediction of cardiovascular effects is warranted. To date, several antidiabetic compounds have been shown to have beneficial effects on the vasculature, independently from their glucose lowering capacity.

#### 3.1.1. Metformin

Metformin is the first-line hypoglycemic drug in T2D. Its cardiovascular benefits have been reported in various clinical trials (reviewed in [88]) and experimental studies have indicated protective effects in ischemic myocardial injury [88]. Preclinical studies suggesting antiatherogenic effects of metformin have mainly been limited to studies on rabbits performed in the 1970s (reviewed in [89]). Studies in mice are sparse, maybe due to the ~15 times higher doses (400 mg/kg/day) required in mice to reach similar therapeutic plasma levels as those in patients taking 2 g of metformin per day [90], increasing the risk of side effects and misinterpretation of results. In addition, the efficacy of metformin in mice seems to vary considerably. In STZ-induced diabetic ApoE^−/−^ mice, metformin failed to control blood glucose and reduce atherosclerosis [91].

#### 3.1.2. Peroxisome Proliferator-Activated Receptor Gamma Activators

The cardiovascular effects of peroxisome proliferator-activated receptor gamma (PPAR*γ*) activators have been more extensively studied in atherosclerotic models. PPAR*γ* agonists are insulin sensitizers, which improve glycemic control and reduce dyslipidemia, leading to antiatherogenic effects. Despite some variability in the lipid-lowering efficacy (partly reflecting the differences between different PPAR*γ* agonists), quite uniform responses with improved lipid profiles and beneficial effects on atherosclerosis have been seen both in mouse and in rabbit models, especially with pioglitazone. The cardiovascular effects of many antidiabetic substances are often seen in nondiabetic animal models, but some effects may only become apparent in the context of diabetes. As an example, some of the lipid-lowering effects of PPAR*γ* agonists, attributable to defects in fatty acid use or storage are only observed in the context of diabetes, but not in nondiabetic atherosclerotic models in which the dyslipidemia is caused, for example, by defective ApoE [92].

Limited information is available on the effects of PPAR*γ* agonists in models that combine diabetes and CVD. In STZ-induced diabetic ApoE^−/−^ mice, rosiglitazone has been shown to have mainly neutral effects on metabolic parameters, but yet to decrease atherosclerosis [93]. On the other hand, in the ob/ob/LDLR^−/−^ApoB^100/100^ mice, rosiglitazone improved the diabetic parameters but worsened hyperlipidemia and atherosclerosis [94]. Pioglitazone, which compared to rosiglitazone has a more favorable effect on lipid profile, reduced the lipid contents of aortic lesions without decreasing the extent of atherosclerosis in insulin resistant ApoE^−/−^ mice (IRS2^+/−^ApoE^−/−^ mice) [42]. However, since the metabolic parameters and the effect on insulin sensitivity were not reported, the overall treatment effect remains elusive. Due to the significant variability between different models, it is difficult to draw conclusions about the backtranslation of PPAR*γ* agonists in current animal models, but in general these compounds have been shown to elicit antiatherogenic effects.

#### 3.1.3. Incretin-Based Therapies

Following the increasing amount of clinical data around the beneficial cardiovascular effects of incretin mimetic drugs and dipeptidyl peptidase-4 (DPP-4) inhibitors, corresponding preclinical research in animal models has markedly increased. So far, nondiabetic LDLR^−/−^ [95] and CETP-ApoB100 transgenic mice [96] have been found to reproduce the beneficial cardiovascular effects of incretin-based therapies described in clinical studies. In ApoE^−/−^ mice, similar findings have been made in many [97–102], but not all studies [91]. While most of the evidence so far is from studies using nondiabetic ApoE^−/−^, there is limited data on the effects of incretin-based therapies on atherosclerosis in diabetic ApoE^−/−^ mice. One study using the glucagon-like peptide 1 (GLP-1) analog taspoglutide reported improvement of diabetic parameters and reduced hepatic lipid accumulation but no effects on atherosclerosis [91]. Another study reported improvement of both hyperglycemia and atherosclerosis after a long-term treatment with the DPP-4 inhibitor alogliptin [102]. Given that DPP-4 is identical to CD26, a cell surface glycoprotein with multiple functions in T cell activation, DNA synthesis, cell proliferation, and cytokine production, it was suggested that DPP-4 inhibitors might affect atherosclerosis by virtue of immune modulation [102]. However, an elegant study comparing the effects of the GLP-1 receptor blocker, exendin (9-39), the glucose-dependent insulinotropic polypeptide (GIP) receptor blocker (Pro(3))GIP, or saline, each coinfused with the DPP-4 inhibitor vildagliptin, demonstrated that the antiatherosclerotic effects of vildagliptin in both diabetic and nondiabetic mice were mainly, but not completely, attributable to the action of the two incretins [103].

An important observation was made in a study where GIP infusion was described to significantly increase hyperglycemia and body weight in ApoE^−/−^ mice with STZ-induced diabetes, but not in spontaneously diabetic* db/db* mice [104], suggesting that GIP might actually modulate the effects of STZ. Moreover, despite this diabetic effect, GIP treatment still reduced the lesion development in the diabetic ApoE^−/−^ mice, indicating that the antiatherogenic mechanism was separate from the effects on glucose metabolism. These findings highlight the importance of understanding the model-specific characteristics before translating observations into general conclusions.

In summary, backtranslation of clinically used antidiabetes agents is far from optimal but has revealed additional cardiovascular effects unrelated to their glucose lowering capacity. Some of the contradictory results may be avoided in the future by a more rational selection of the animal models. As an example, most of the abovementioned studies have been performed using STZ, yielding insulinopenic and insulin sensitive mice. Despite being a standard model in preclinical research, it does not optimally reflect the clinical picture of T2D. Given that some insulin secretion capacity is needed for most antidiabetes agents to work, the STZ protocol must be carefully adjusted to maintain sufficient number of functional *β*-cells. Also, for the evaluation of insulin sensitizing drugs, the presence of insulin resistance is needed. It is therefore important to extend the benchmarking and validation to cover also other available models, in addition to developing new ones. And as the key for selecting the appropriate models for these studies, a better understanding of how insulin resistance and T2D promote atherogenesis and plaque progression is essential [105].

### 3.2. Antiatherosclerotic Agents in Atherosclerotic Models

#### 3.2.1. Lipid-Lowering Agents

Responses to interventions treating hypercholesterolemia, hypertriglyceridemia, hypertension, and inflammation in different hyperlipidemic mouse models have been thoroughly reviewed by Zadelaar et al. [106]. In brief, the lipid-lowering effects of statins (3-hydroxy-3-methylglutaryl coenzyme A reductase inhibitors) and the subsequent effects on atherosclerosis seem variable in ApoE^−/−^ and LDLR^−/−^ mice. In contrast, the effects of statins in ApoE^*^3-Leiden mice are more uniform and human-like. Effects of fibrates (PPAR*α* agonists) appear to be slightly more similar in different models, although the degree of lipid-dependence of these antiatherosclerotic effects is unclear. Of all lipid-lowering agents, ezetimibe, which acts through inhibition of cholesterol uptake and absorption in the intestine, shows the most consistent hypolipidemic and antiatherosclerotic effects in mice. However, this does not fully translate to clinical trials, which show inconsistent effects of ezetimibe on disease outcomes, despite clear cholesterol-lowering efficacy [107].

#### 3.2.2. Hypotensive Agents

Among the hypotensive agents, blocking the effects of angiotensin II (Ang II) via angiotensin-converting enzyme (ACE) inhibitors or Ang II type 1 (AT_1_) receptor antagonists has in most studies resulted in decreased atherosclerosis [108]. Also, calcium channel blockers have been reported to suppress the progression of atherosclerosis in ApoE^−/−^ mice [109, 110]. However, since marked hypertension is usually absent in mouse models, most of the effects of these agents on atherosclerosis are somewhat dissociated from their hypotensive activity.

## 4. Conclusions

Diabetes is a complex disease, and there is no single animal model which can mimic the full spectrum of the human disease. However, there is a broad range of different models that recreate different aspects of macrovascular disease. The selection of the right model for each study and the experimental design are critical, since models can lack mechanisms required for the tested drug to be effective. Several of the models discussed in this review are useful platforms that could be further “tuned up” by genetic manipulation in order to accelerate or exacerbate specific features of the human disease or “tuned down” by introducing potentially protecting genes. A more systematic testing of candidate genes identified by large scale human GWAS studies on these already existing platforms may be a powerful approach to improve the translational value of experimental diabetic research.

## Figures and Tables

**Table 1:** Physiological differences between human, mouse, and rat relevant for the study of diabetic vascular complications.

Characteristic	Human	Mouse	Rat

Life span (years)	~80	~2-3	~2-3
Heart rate (beats/minute)	60–80	>400	~400
Blood glucose (mmol/L)	4.4–6.1	3.5–11.9	3.9–11.5
Blood pressure (mm Hg)	90–119/60–79	113–147/81–106	75–120/60–90
ALT (IU/L)	5–50	20–90	18–45
AST (IU/L)	6–40	90–100	74–143
Plasma cholesterol (mmol/L)	3.0–6.5	1.0–5.3	1.0–3.8
Dominant lipoprotein	LDL	HDL	HDL
Cholesterol synthesis (mg/day/kg)	10 [111]	160 [111]	70 [112]
Hepatic LDL clearance (mL/day/kg)	12 [111]	400–500 [111]	220 [112]
ApoB subtypes in liver	B100	B48 and B100	B48 and B100
Apo(a) expression	Yes	No	No
CETP	Yes	No	No
Major acute phase protein	CRP, SAA	SAA, SAP	A2M, HAPT
Diameter of the atherosclerosis-prone arteries (mm)			
Ascending aorta	29–33 [113, 114]	1.2–1.5 [115]	3.1–3.7 [116]
Left main coronary artery	~4 [114]	0.1–0.16 [54, 117, 118]	0.1–2.4
Carotid artery	6–8 [114]	0.45–0.55 [119, 120]	0.9
Brachiocephalic trunk	~12 [114]	~0.5 [121]	~1.0

A2M, alpha-2-macroglobulin; ALT, alanine aminotransferase; AST, aspartate aminotransferase; Apo, apolipoprotein; CETP, cholesteryl ester transfer protein; CRP, C-reactive protein; HAPT, haptoglobin; HDL, high-density lipoprotein; LDL, low-density lipoprotein; SAA, serum amyloid A; SAP, serum amyloid P. Additional mouse phenotype data can be found at MPD. Values were obtained from the specified references, from the Mouse Phenome Database (MPD; http://phenome.jax.org), and from unpublished data by the authors.

**Table 2:** Atherosclerotic mouse models with increased atherosclerosis after diet-induced diabetes.

Mouse model	Inducer	Hyperlipidemia	Atherosclerosis	Comments	Reference

Diabetes increases atherosclerosis without changes in plasma lipids					
ApoE^−/−^	HFD (17 wks)	TC*↔* (15.6 mM) TG*↔* (1.1 mM)	↑	Glucose↑, IR versus mice w/equal lipids on low-fat diet	[122]
LDLR^−/−^	DD (18–24 wks)	TC*↔* (43.4 mM)	↑ (calcif.)	Glucose↑, IR	[123]

Diabetes increases atherosclerosis and alters plasma lipid levels					
LDLR^−/−^	WD (17 wks)	TC↑ (27.0 mM) TG↑ (4.3 mM)	↑	Glucose↑, insulin↑, versus HFD w/o cholesterol + similar dysglycemia: lesions↑	[124]
LDLR^−/−^	WD (4.5 mos)	TC↑ (25.0 mM) TG↑ (2.6 mM)	↑	Insulin↑, IR; versus fructose diet w/equal cholesterol + euglycemia: lesions*↔*	[125]
LDLR^−/−^	DD (16 wks)	TC↑ (17.9 mM) TG↑ (5.0 mM)	↑	Glucose↑, insulin↑, IR	[126]
ApoE^−/−^	WD (5 wks)	TC↑ (33.8 mM) TG↑ (1.1 mM)	NI↑	Glucose↑, insulin↑	[127]
ApoE^*^3-Leiden	HCD (28 wks)	TC↑ (20 mM) TG↑ (1.7 mM)	↑	IR	[106]
HuB	HFD (12 mos)	TC↑ (3.5 mM) TG↑ (1.0 mM)	Fatty streaks	Glucose↑, insulin↑, IR	[128]
ApoE^−/−^	Fructose (8 wks)	TC*↔* (9.9 mM) TG↑ (2.1 mM)	↑	Glucose↑, insulin↑	[129]

DD, diabetogenic diet (Bioserve F1850: 35.5% energy from fat); HCD, high-cholesterol diet (20% energy from fat, 0.15–1.25% cholesterol); HFD, high-fat diet (20–60% energy from fat, usually no cholesterol); IR, insulin resistance; NI, neointima; TC, total cholesterol; TG, triglycerides; WD, Western diet (Harlan Teklad TD96125 or TD88137).

**Table 3:** Atherosclerotic mouse models with increased atherosclerosis after chemically induced diabetes.

Mouse model	Inducer	Hyperlipidemia	Atherosclerosis	Comments	Reference

Diabetes increases atherosclerosis without changes in plasma lipids					
ApoE^−/−^	STZ	TC*↔* (9.1 mM) TG*↔* (1.3 mM)	↑ × 2	Calcified lesions	[130]
ApoE^−/−^GPx1^−/−^	STZ	TC*↔* (13.8 mM) TG*↔* (2.1 mM)	↑ × 4		[131]
LDLR^+/−^	STZ + CCA (12 wks)	TC*↔* (14.1 mM) TG*↔* (0.6 mM)	↑	Cholic acid	[132]
LDLR^+/−^hAR	STZ + CCA (12 wks)	TC*↔* (13.6 mM) TG*↔* (0.6 mM)	↑ × 2	Cholic acid; versus LDLR^+/−^ + STZ: lipids*↔*, lesions↑ × 2	[132]
ApoE^−/−^hAR	STZ	TC*↔* (31.1 mM) TG*↔* (3.1 mM)	↑ × 2		[133]

Diabetes increases atherosclerosis and alters plasma lipid levels					
Balb/c	STZ + AD (12–20 wks)	TC↑ (9.8 mM) TG↑ (0.6 mM)	↑ × 17	Cholate	[134]
ApoE^−/−^	STZ	TC↑ (35.3 mM) TG*↔* (1.7 mM)	↑ × 5	sRAGE → lesions ↓	[135]
LDLR^−/−^	STZ + WD (6 wks)	TC↑ (44.3 mMl) TG↑ (6.3 mM)	↑ × 6.5		[136]
HuB/LPL^+/−^	STZ + WD (20 wks)	TC↑ (13.8 mM) TG↑ (5.5 mM)	↑ × 14		[137]
LDLR^−/−^Apobec-1^−/−^	STZ	TC↑ (9.9 mM) TG*↔* (1.6 mM)	↑ × 1.5		[138]
LDLR^−/−^	STZ + HCD (12 wks)	TC↑ (91.6 mM) TG*↔* (1.3 mM)	↑ × 3		[132]
LDLR^−/−^hAR	STZ + HCD (12 wks)	TC↑ (95.2 mM) TG*↔* (1.0 mM)	↑ × 4	Versus LDLR^−/−^ + STZ: lipids*↔*, lesions↑ × 2	[132]

AD, atherogenic diet (12.5–40% energy from fat, 0.075–1.5% cholesterol, possibly with 0.5% sodium cholate); CCA, cholesterol/cholic acid–containing diet (1.25% cholesterol and 0.5% sodium cholate); HCD, high-cholesterol diet (20% energy from fat, 0.15–1.25% cholesterol); sRAGE, soluble receptor for AGEs; STZ, streptozotocin; TC, total cholesterol; TG, triglycerides; WD, Western diet (Harlan Teklad TD96125 or TD88137).

**Table 4:** Atherosclerotic mouse models with increased atherosclerosis after genetically induced diabetes.

Mouse model	Diet	Hyperlipidemia	Atherosclerosis	Comments	Reference

Diabetes increases atherosclerosis without changes in plasma lipids					
LDLR^−/−^GP		TC*↔* (8.8 mM) TG*↔* (2.1 mM)	↑ × 3		[139]
ApoE^−/−^IRS2^−/−^	WD (9–12 wks)	TC*↔* (64.9 mM) TG*↔* (0.8 mM)	↑		[140]
ApoE^−/−^IRS2^−/−^	AD (4 wks)	TC*↔* (15.6 mM)	↑		[141]
ApoE^−/−^Insr^+/−^Irs1^+/−^	WD (15 wks)	TC*↔* (24.0–30.9 mM) TG*↔* (1.1–1.5 mM)	↑		[44]

Diabetes increases atherosclerosis and alters plasma lipid levels					
LDLR^−/−^ob/ob		TC↑ (44.5 mM) TG↑ (11.5 mM)	↑		[38]
LDLR^−/−^GP	AD (12 wks)	TC↑ (34.9 mM) TG↑ (23.8mM)	↑ × 2	Lesion hemorrhages↑	[139]
LDLR^−/−^ob/ob		TC↑ (17.2 mM) TG↑ (3.4 mM)	↑ × 4		[36]
LDLR^−/−^ApoB^100/100^ob/ob		TC↑ (44.8 mM) TG↑ (7.4 mM)	↑	Versus C57Bl/6: Insulin↑, BP↑	[39]
ApoE^−/−^db/db		TC↑ (15.1 mM) TG↑ (2.6 mM)	↑ × 3		[37]
ApoE^−/−^db/db	WD (10 wks)	TC↑ (37.9 mM) TG↑ (10.1 mM)	↑		[37]
ApoE^−/−^db/db		TC↑ (24.8 mM) TG*↔* (0.2 mM)	↑ × 3		[142]
ApoE^−/−^db/db	Chow diet	TC↑ (30–40 mM) TG*↔* (1-2 mM)	↑ × 3-4	C57BL/6 background	∗
ApoE^−/−^ob/ob		TC↑ (17.7 mM) TG*↔* (1.5 mM)	↑ × 3		[36]
ApoE^−/−^ApoB^100/100^ob/ob		TC↑ (31.2 mM) TG↑ (3.8 mM)	↑	Versus C57Bl/6: glucose↑, insulin↑, IR, BP↑	[39]
LDLR^−/−^Ins2^Akita^	HCD (16 wks)	TC↑ (32.5 mM) TG↑ (5.3 mM)	↑ × 2	Phenotype stronger in males	[25]
ApoE^−/−^Ins2^Akita^		TC↑ (22.5 mM) TG↑ (0.9 mM)	↑ × 3		[24]
E4hLDLR-tg Ins2^Akita^		TC↑ (2.6 mM) TG*↔* (0.5 mM)	↑	Non-HDL-C/HDL-C ratio~3	[143]
KKAy^+^ApoE^−/−^		TC↑ (7.6 mM) TG↑ (1.3 mM)	↑		[41]
Trail^−/−^ApoE^−/−^	WD (12 wks)	TC↑ (23 mM) TG↑ (2.2 mM)	↑		[144]

AD, atherogenic diet (10.8–40% energy from fat, 0.075–1.5% cholesterol, possibly with 0.5% sodium cholate); BP, blood pressure; E4, human ApoE^*^4 allele; HCD, high-cholesterol diet (20% energy from fat, 0.15–1.25% cholesterol); hLDLR, human LDLR; TC, total cholesterol; TG, triglycerides; WD, Western diet (Harlan Teklad TD96125 or TD88137). ^*^Hübschle T, Hiss K. unpublished data.
